# Clinical effect of reduning combined with gamma globulin treatment on symptom improvement serum levels of IL-6, 25-(OH)D and LDH in children with severe mycoplasma pneumonia

**DOI:** 10.12669/pjms.38.4.5203

**Published:** 2022

**Authors:** Xingna Ma, Wei Gao, Jinyu An

**Affiliations:** 1 Xingna Ma, Department of Pediatric, Changzhou Cancer Hospital Affiliated to Soochow University, Changzhou, 213000, Jiangsu, China; 2 Wei Gao, Department of Pediatric, Changzhou Cancer Hospital Affiliated to Soochow University, Changzhou, 213000, Jiangsu, China; 3 Jinyu An, Department of Orthopedics, Changzhou Cancer Hospital Affiliated to Soochow University, Changzhou, 213000, Jiangsu, China

**Keywords:** Severe mycoplasma pneumonia, Gamma globulin, Reduning, Symptom improvement, IL-6, LDH, 25-(OH)D

## Abstract

**Objectives:**

To investigate the effects of Reduning combined with gamma globulin on symptom improvement and serum levels of interleukin-6 (IL-6), 25-hydroxyvitamin D[25-(OH)D] and lactate dehydrogenase (LDH) in children with severe mycoplasma pneumonia (MP).

**Methods:**

A total of 123 children with severe MP admitted to the Changzhou Cancer Hospital affiliated to Soochow University from May 2018 to April 2020 were selected as subjects and randomly divided into three groups: control Group-A, control Group-B and the observation group, with 41 cases in each group. Control Group-A was given with Reduning, control Group-B was treated with gamma globulin, while the observation group was treated with Reduning injection combined with gamma globulin. The clinical efficacy, symptom improvement, incidence of adverse reactions, serum levels of IL-6, 25-(OH)D, LDH, Toll-like receptor 4/myeloid differentiation factor 88 (TLR4/MyD88) signaling pathway and T lymphocyte subsets before and after treatment were statistically compared between the three groups.

**Results:**

The total effective rate of treatment in the observation group was higher than that in control Group-A and control Group-B (*p<*0.05); The time for body temperature, lung rales, and cough to return to normal in the observation group was shorter than that in control Group-A and control Group-B (*p<*0.05). After treatment, the observation group had lower serum levels of IL-6, LDH, and higher levels of 25-(OH)D compared with control Group-A and control Group-B (*p<*0.05). Moreover, the observation group had lower serum expressions of TLR4 and MyD88 (*p<*0.05), higher serum levels of CD4^+^, CD4^+^/CD8^+^, and lower CD8^+^ compared with control Group-A and control Group-B (*p<*0.05). No significant difference can be seen in the comparison of the incidence of adverse reactions between the three groups (*p>*0.05).

**Conclusions:**

Reduning combined with gamma globulin in the treatment of severe MP is worthy of clinical promotion and application. With such a treatment regimen, the immune function of children with severe MP can be significantly enhanced, the inflammatory response can be suppressed, and symptom improvement can be promoted, further improving the treatment outcome.

## INTRODUCTION

Mycoplasma pneumonia (MP) is a common pathogen that gives rise to respiratory infectious diseases such as pediatric pneumonia^1^. MP occurs frequently in preschool children and may induce rhinitis, otitis media, tonsillitis, etc^2^, seriously affecting the physical and mental health of children.^3^ Antibacterial agents have been clinically preferred for the treatment of mycoplasma pneumonia, but antibacterial agents alone have not achieved the desired effect. With the improvement of clinical research on the pathogenesis of MP, immunopathogenesis is believed to play a critical role in the pathogenesis of the disease.^4^

Reduning, as a pure traditional Chinese medicine preparation, boasts of enhancing the body’s immune function in addition to its heat-clearing, anti-inflammatory and anti-pathogen effects.^5^ Gamma globulin presents a broad-spectrum antibacterial and antiviral effect, which can stimulate the passive immune function of the human body, thereby alleviating the damage to the body caused by virus infection.^6^ Based on this, in this study, Reduning and gamma globulin were combined to treat severe MP, and its effects on symptoms improvement and serum levels of interleukin-6 (IL-6), 25-hydroxyvitamin D[25-(OH)D] and lactate dehydrogenase (LDH) were analyzed.

## METHODS

A total of 123 children with severe MP admitted to the Changzhou Cancer Hospital affiliated to Soochow University from May 2018 to April 2020 were randomly selected as subjects after being examined and approved by the Medical Ethics Committee of the Hospital.

### Ethical Approval:

The study was approved by the Institutional Ethics Committee of Changzhou Cancer Hospital affiliated to Soochow University on May 13, 2018(No.:201832), and written informed consent was obtained from all participants.

### Inclusion Criteria:

- Children who meet the relevant diagnostic criteria of MP in “Practical Pediatrics”and have been diagnosed by serum MP examination and imaging examination;
- Children who have given informed consent to this study and voluntarily signed relevant documents by themselves and their legal guardians.

### Exclusion Criteria:

- Children with respiratory failure and heart failure;
- Children with primary diseases such as heart, liver and kidney, immune system, hematopoietic system;
- Children who are treated with antibiotics or other immunosuppressive agents before admission;
- Children with allergies or contraindications to the drugs used in this study.

All children were randomly divided into three groups with 41 cases in each group. The clinical data [gender, age, course of disease, body temperature, white blood cell count (WBC), and imaging findings] of the three groups were equably comparable (*p*>0.05). Table-I.

**Table-I:** Comparison of clinical data of the three groups.

Data	Observation group (n=41)	Control Group-A (n=41)	Control Group-B (n=41)	t/χ^2^	P
*Gender:* (Male/Female)	24/17	26/15	23/18	0.472	0.790
Age (years old)	1-12 (6.45±2.73)	1-13 (6.94±3.01)	2-13 (7.52±2.82)	1.442	0.240
Course of disease	1-7 (4.18±1.56)	2-7 (4.47±1.39)	2-8 (4.92±1.58)	2.492	0.087
Body temperature	38.2-39.4 (38.82±0.31)	38.1-39.6 (38.85±0.37)	38.4-39.5 (38.95±0.35)	1.603	0.206
WBC count (×10^9^/L)	10-12 (11.12±0.56)	11-12 (11.24±0.37)	10-13 (11.33±0.62)	1.635	0.199
*Imaging findings*				0.712	0.950
Lung parenchyma	21 (51.22)	20 (48.78)	22 (53.66)		
Atelectasis	8 (19.51)	10 (24.39)	7 (17.07)		
Pleural effusion	12 (29.27)	11 (26.83)	12 (29.27)		

All the three groups were treated with conventional treatments such as cough, phlegm, fever, oxygen inhalation, supplemented with azithromycin treatment. Specifically, 10 mg/kg azithromycin for injection (Pharmacia & Upjohn Company LLC, J20140073) was mixed with 250 mL normal saline for intravenous infusion, once a day for 5 consecutive days. Subsequently, azithromycin dispersible tablets (Zhejiang Yatai Pharmaceutical Co., Ltd., State Drug Approval No.: H10980289) were given orally at 5 mg/(kg·d) once a day. Control Group-A was given 0.5 mg/kg Reduning injection (Jiangsu Kanion Pharmaceutical Co., Ltd., State Drug Approval No.: Z20050217) mixed with 250 mL normal saline for intravenous infusion, once a day. Control Group-B was intravenously injected with gamma globulin (Sichuan Yuanda Shuyang Pharmaceutical Co., Ltd.) combined with 400 mg/kg azithromycin for injection and 250 mL normal saline, once a day. The observation group was treated with Reduning combined with gamma globulin, and the usage and dosage were the same as those in control groups A and B. The three groups were treated continuously for seven days.

About 5-mL of cubital venous blood was extracted from all the three groups under fasting condition, and centrifuged at a high speed (3000 r/min, 10 minutes). After centrifugation, the serum was collected for examination. The serum IL-6 level was determined by enzyme-linked immunosorbent asaay (ELISA), the serum 25-(OH)D level was determined by electrochemiluminescence immunoassay (ECLIA), and the serum LDH level was determined by enzymatic reaction method. The kits were purchased from Beijing Hua Ketai Biotechnology Co., Ltd.; The expressions of serum TLR4 and MyD88 were determined by Western blot, the gray value ratio of target protein strip to β-actin strip was used to reflect the expression of the target protein. The kits were purchased From Santa Crut, USA; T lymphocyte subsets were determined by flow cytometry provided by Beckman, USA, and the operation was strictly in accordance with the kit and instrument instructions.

### Clinical efficacy, efficacy criteria:

Cured: Disappearance of symptoms and signs, normal X-ray examination, normal laboratory indicators such as WBC, neutrophils (NE) and red blood cell count (RBC); Markedly effective: Basically disappearance of symptoms and signs, disappearance of most of the X-ray shadow, one abnormal laboratory indicator; Effective: Improvement of symptoms and signs, shrink of X-ray shadow, no or one normal laboratory indicator; Invalid: Failure to meet the above criteria. Total effective rate = (cured + markedly effective + effective)/41×100%.

Symptom improvement, including the time for body temperature, lung rales, and cough to return to normal. Serum levels of IL-6, 25-(OH)D and LDH before and after treatment. Factors related to TLR4/MyD88 signaling pathway before and after treatment. Peripheral blood T lymphocyte subsets before and after treatment. Incidence of adverse reactions, including nausea, vomiting, diarrhea, discomfort, etc.

### Statistical Analysis

All the data were statistically analyzed by SPSS21.0 software, and the measurement data were expressed as (x̄±s) using t test. Counting data were expressed by n(%) using χ2 test; One-way ANOVA was used for comparison between multiple groups, and LSD-t test was used for pairwise comparison. P<0.05 indicates a statistically significant difference.

## RESULTS

The total effective rate of the observation group was higher than that of control groups A and B (*p*<0.05), with no significant difference in the total effective rate of control groups A and B (*p*>0.05). Table-II.

**Table-II:** Comparison of clinical efficacy of the three groups [n (%)].

Group	Number of cases	Cured	Markedly effective	Effective	Invalid	Total effective
Observation group	41	16 (39.02)	12 (29.27)	11 (26.83)	2 (4.88)	39 (95.12)
Control Group-A	41	10 (24.39)	9 (21.95)	13 (31.71)	9 (21.95)	32 (78.05)
Control Group-B	41	9 (21.95)	10 (24.39)	12 (29.27)	10 (24.39)	31 (75.61)
χ^2^						6.546
P						0.038

The time for body temperature, lung rales and cough to return to normal in the observation group was shorter than that in control groups A and B (*p*<0.05), with no significant difference in the time for body temperature, lung rales and cough to return to normal in control groups A and B (*p*>0.05). Table-III.

**Table-III:** Comparison of symptom improvement in the three groups (x¯ ±s, d).

Group	Number of cases	Time for body temperature to return to normal	Time for lungs rales to return to normal	Time for cough to return to normal
Observation group	41	4.52±1.41	5.21±1.37	6.21±1.64
Control Group-A	41	6.27±1.63	7.35±2.28	9.32±1.47
Control Group-B	41	6.84±1.69	7.74±2.35	9.85±1.56
F		23.967	18.116	65.300
P		<0.001	<0.001	<0.001

After treatment, the serum levels of IL-6 and LDH in the observation group were lower, and the levels of 25-(OH)D were higher than those in control groups A and B (*p*<0.05), with no significant difference in the serum levels of IL-6 and LDH in control groups A and Group-B (*p*>0.05). Table-IV.

**Table-IV:** Comparison of serum levels of IL-6, 25-(OH)D and LDH in the three groups (x¯ ±s)

Group	Number of cases	IL-6 (pg/ml)		25-(OH)D (ng/ml)		LDH (IU/L)	

		Before treatment	After treatment	Before treatment	After treatment	Before treatment	After treatment
Observation group	41	80.16±20.47	14.18±4.13	18.84±2.69	29.74±3.52	288.47±35.12	101.35±9.52
Control Group-A	41	85.24±22.31	20.85±8.62	19.12±2.73	26.93±3.15	284.39±37.62	134.28±9.71
Control Group-B	41	83.17±20.85	21.37±8.74	18.54±2.58	25.87±3.09	287.94±35.14	136.28±9.86
F		0.594	11.788	0.485	15.440	0.156	167.736
P		0.554	<0.001	0.617	<0.001	0.856	<0.001

After treatment, the serum expressions of TLR4 and MyD88 in the observation group were lower than those in control groups A and B (*p*<0.05), with no significant difference in the serum expressions of TLR4 and MyD88 between control groups A and B (*p*>0.05). Table-V. After treatment, the serum levels of CD4^+^ and CD4^+^/CD8^+^ in the observation group were higher than those in control groups A and B, and CD8^+^ were lower than those in control groups A and B (*p*<0.05), with no significant differences in the serum levels of CD4^+^, CD8^+^ and CD4^+^/CD8^+^ between control groups A and B (*p*>0.05). Table-VI. There was no significant difference in the incidence of adverse reactions between the three groups (*p*>0.05). Table-VII.

**Table-V:** Comparison of factors related to TLR4/MyD88 signaling pathway in the three groups (x¯ ±s).

Group	Number of cases	TLR4		MyD88	

		Before treatment	After treatment	Before treatment	After treatment
Observation group	41	1.54±0.12	0.20±0.04	1.29±0.10	0.17±0.05
Control Group-A	41	1.58±0.14	0.45±0.07	1.33±0.12	0.37±0.12
Control Group-B	41	1.56±0.12	0.47±0.09	1.30±0.12	0.40±0.15
F		1.017	190.678	1.374	48.805
P		0.365	<0.001	0.257	<0.001

**Table-VI:** Comparison of T lymphocyte subsets in the three groups (x¯±s).

Group	Number of cases	CD4^+^ (%)		CD8^+^ (%)		CD4^+^/CD8^+^	

		Before treatment	After treatment	Before treatment	After treatment	Before treatment	After treatment
Observation group	41	21.12±1.75	28.67±3.70	17.83±1.83	13.62±2.23	1.18±0.17	2.10±0.37
Control Group-A	41	21.52±2.04	25.27±3.25	17.43±1.85	15.56±2.01	1.23±0.20	1.62±0.32
Control Group-B	41	21.41±1.84	24.69±3.31	17.70±1.81	15.82±2.14	1.21±0.18	1.55±0.34
F		0.495	16.150	0.510	13.078	0.769	31.065
P		0.611	<0.001	0.602	<0.001	0.466	<0.001

**Table-VII:** Comparison of the incidence of adverse reactions in the three groups [n (%)].

Group	Number of cases	Nausea, vomiting	Diarrhea	Discomfort	Total incidence
Observation group	41	3 (7.32)	2 (4.88)	2 (4.88)	7 (17.07)
Control Group-A	41	3 (7.32)	1(2.44)	1 (2.44)	5 (12.20)
Control Group-B	41	1 (2.44)	0()	2 (4.88)	3 (7.32)
χ^2^					1.822
P					0.402

## DISCUSSION

With the increasing proportion of MP in pediatric pneumonia, the number of cases of severe MP is also on the rise, which has attracted great clinical attention.^7^ Severe MP is characterized by rapid disease progression, systemic inflammatory response syndrome, extrapulmonary complications, and severe pulmonary disease. It is therefore particularly critical to take effective and safe treatment of severe MP in a timely manner.^8^

Antimicrobial agents such as azithromycin are currently preferred in the clinical treatment of severe MP. However, long-term azithromycin administration may give rise to high drug resistance, and can cause adverse reactions such as vomiting, diarrhea, and pain at the injection site, affecting the health of children.^9^ Immune dysfunction is an important factor in the pathogenesis of MP.^10^ Based on this, in this study, Reduning and gamma globulin were combined to treat children with severe MP. The results showed that the time for body temperature, lung rales and cough to return to normal in the observation group was shorter than that in control groups A and B, and the total effective rate was higher than that in control groups A and B, suggesting that the combined treatment of Reduning and immune globulin can improve the symptoms of children with severe MP and further improve the treatment outcome. Gamma globulin can treat severe MP because of the following:

### Anti-infective effect:

Gamma globulin boasts of rapidly increasing the level of immune globulin G in the blood, neutralizing toxins with pathogens, and thereby improving symptoms; *Anti-inflammatory effect:* Immune globulin can inhibit the maturation and proliferation of immature T cells, thereby inhibiting the secretion and release of inflammatory mediators and cytokines. The Fc segment of immune globulin G produced in large quantities can bind to the Fc receptor of phagocytes, making it unable to bind to autoantibodies and corresponding cytokines, and blocking the activation of phagocytes, so that the body tissues and cells are protected from damage.

### Immune regulation:

Gamma globulin can feedback inhibit antibody production, regulate immune function, and enhance the body’s ability to fight infection.^11^ MP falls into the category of “pneumonia with dyspnoea and cough” in traditional Chinese medicine, which is mainly attributed to the occlusion of lung collaterals and impaired lung depuration caused by the invasion of the pathogenic factors such as wind pathogen, heat-toxicity and phlegm heat. The treatment of MP should be based on clearing and purging the lung fire, and facilitating the flow of the lung-qi and dredging collaterals. Reduning is a traditional Chinese medicine preparation composed of Artemisia annua, Gardenia jasminoides, and Honeysuckle. Artemisia annua has the effect of ventilating and dissipating heat; Gardenia jasminoides helps to purge fire and eliminate irritability, cool blood and detoxify; Honeysuckle has the effect of clearing heat and detoxicating. The combination of the three medicines is beneficial to clear away the lung fire, facilitate the flow of the lung-qi and dredge collaterals, and remove the evils from the outside and inside. It has been confirmed by modern pharmacology that the honeysuckle in Reduning contains volatile oil, organic acids, flavonoids and other components, with antipyretic, anti-inflammatory, broad-spectrum antibacterial and immune-regulating effects. Artemisinin, as the main component of Artemisia annua, can inhibit B lymphocytes; Geniposide in Gardenia has significant anti-inflammatory and antibacterial effects, and can inhibit edema and exudation in the early stage of inflammation.^12^ Immunoglobulin and Reduning have different mechanisms of action, and the combined application of the two is conducive to synergistic supplementation to further improve the treatment outcome.

Great attention has been placed on the pathogenesis of MP immunology, especially the role of cellular immune mechanisms in the pathogenesis of severe MP.^13^,^14^ In the cellular immune response, CD4^+^ represents the helper induced T cell subsets, and its increase indicates the increase of immune globulin produced by B cells and the enhancement of cellular immunity; CD8^+^ is an inhibitory cell/cytotoxic cell subgroup, and its increase indicates immunosuppression; CD4^+^ and CD8^+^ are inter-conditioned. Under normal circumstances, the ratio of CD4^+^/CD8^+^ is in a dynamic balance, which is an important indicator of the environmental stability in the human immune system. After the onset of MP, CD4^+^ in the body decreases, CD8^+^ increases, and the ratio of CD4^+^/CD8^+^ is in an unbalanced state.^15^ IL-6, as an important cytokine in the inflammatory response of MP, boasts a regulatory effect on cellular and humoral immunity, and exerts its unique effects as early as the early stage of the immune response. IL-6 levels rise sharply after the occurrence of mycoplasma pneumonia, contributing to inducing T cell differentiation, promoting the release of inflammatory cytokines, and aggravating the disease.^16^ 25-(OH)D is an important indicator of vitamin D metabolism in the body, and its deficiency is believed to be associated with a variety of immune diseases, such as type-I diabetes, asthma, severe pneumonia. 25-(OH)D, as a neuroendocrine-immunomodulatory hormone, can affect the body’s inflammatory response and antimicrobial activity by regulating the balance of calcium and phosphorus, acting on neutrophils, macrophages, lymphocytes and respiratory epithelial cells, and stimulating the expression of antimicrobial polypeptides, which plays an important role in pulmonary infection.

It has been shown in related studies that there is a certain correlation between 25-(OH)D and T cell subsets in children with MP. LDH is a glycolytic enzyme found in the cytoplasm of all tissue cells in the body. In case of severe damage to the lung tissue, LDH isoenzyme in the lung tissue may give rise to the increase of LDH level in blood. It has been confirmed in some studies that LDH is significantly correlated with IL-18. IL-18 is a typical Th1-type cell, which can stimulate the Th1-mediated immune response and has close bearing on the severity of MP.^17^ In this study, the observation group had higher serum levels of CD4^+^, CD4^+^/CD8^+^, 25-(OH)D, as well as lower CD8^+^, IL-6, LDH than control groups A and B after treatment (*p*<0.05), suggesting that Reduning combined with gamma globulin in the treatment of severe MP can significantly enhance the body’s immune function and inhibit inflammation. To explain it, immune globulin has multivalent antigen-specific immunoglobulin antibodies, which can play a dual role in immune substitution and immune regulation, while Reduning can promote the repair of immune function and inhibit inflammation. The combined application of the two is conducive to further enhancing the immune function of the body.

The pathogenic mechanism of MP infection is relatively complex, which is mostly believed to be related to the direct damage of MP to respiratory epithelial cells, immune mechanism and lung inflammatory injury caused by cytotoxic effect.^18^ MP lacks a cell wall, and its inflammatory response has a close bearing on lipid associated membrane proteins on the cell membrane.^19^ It has been confirmed in some studies that lipid-related membrane proteins are TLR ligands, which can recruit downstream signaling molecules MyD88 by binding to TLRs on the surface of monocytes and macrophages. In this way, the protein kinase itself will be phosphorylated to promote the activation and translocation of nuclear factors into the nucleus, thereby inducing mononuclear macrophages to synthesize and secrete inflammatory cytokines, resulting in lung injury.^20^ In this study, the serum levels of TLR4 and MyD88 in the observation group were lower than those in control groups A and B after treatment. When Reduning combined with gamma globulin was used to treat severe MP, the inflammatory response of the body was effectively controlled, the lung injury was alleviated, and the serum expressions of TLR4 and MyD88 were decreased. In addition, no significant difference was observed in the incidence of adverse reactions among the three groups, suggesting that Reduning combined with gamma globulin in the treatment of severe MP would not increase the incidence of adverse reactions.

### Limitation of the study:

It includes the small sample size included in this study. In view of this, clinical multi-center, studies with large sample size are needed to confirm our observations.

## CONCLUSIONS

Reduning combined with gamma globulin in the treatment of severe MP is worthy of clinical promotion and application. With such a treatment regimen, the immune function of children with severe MP can be significantly enhanced, the inflammatory response can be suppressed, and symptom improvement can be promoted, further improving the treatment outcome.

### Authors’ Contributions:

**XM & JA:** Designed this study and prepared this manuscript,and are responsible and accountable for the accuracy or integrity of the work.

**XM & JA:** Collected and analyzed clinical data.

**WG:** Significantly revised this manuscript.
